# Higher Awareness of Positive and Negative Age-Related Changes Relate to Lower Objective Cognitive Scores

**DOI:** 10.1093/geroni/igaa057.2010

**Published:** 2020-12-16

**Authors:** Serena Sabatini, Obioha Ukoumunne, Clive Ballard, Kaarin Anstey, Manfred Diehl, Allyson Brothers, Hans-Werner Wahl, Linda Clare

**Affiliations:** 1 University of Exeter, Exeter, England, United Kingdom; 2 University of New South Wales, Sydney, Australia; 3 Colorado State University, Fort Collins, Colorado, United States; 4 University of Heidelberg, Heidelberg, Baden-Wurttemberg, Germany

## Abstract

Existing evidence suggests that individuals’ subjective experience of cognitive decline may be a risk state for dementia. However, whether self-awareness of positive changes confer cognitive protection is unknown. We examined the extent to which awareness of positive (AARC gains) and negative (AARC losses) age-related changes explains variability in objective cognitive performance in a sample of 6,231 UK residents (Mean age= 66.1 years, 75.9% women) without cognitive impairment. We tested a structural equation model with AARC gains and losses as predictors of cognitive performance and depressive symptoms as a mediator of the association of AARC losses with cognitive performance. The model fit the data well. The correlation between AARC gains and losses was negligible, yet higher levels of both AARC gains and losses predicted poorer cognitive scores. Hence, higher AARC gains did not confer cognitive protection. This unexpected pattern of results underscores the complexity of mapping individuals’ awareness onto objective outcomes.

